# Potential pro‐tumour cytokine in oral squamous cellular carcinoma: IL37

**DOI:** 10.1111/jcmm.70167

**Published:** 2024-11-05

**Authors:** Ying Yan, Jun Li, Yungang He, Ping Ji, Jie Xu, Yong Li

**Affiliations:** ^1^ Stomatological Hospital of Chongqing Medical University Chongqing China; ^2^ Chongqing Key Laboratory for Oral Diseases and Biomedical Science Chongqing China; ^3^ Chongqing Municipal Key Laboratory of Oral Biomedical Engineering of Higher Education Chongqing China

**Keywords:** GSDMD, IL37, macrophage, NLRP3, OSCC

## Abstract

Inflammation and immunosuppression are important features of tumours, including oral squamous cellular carcinoma (OSCC). Interleukin 37 (IL37), a cytokine known for the ability to suppress inflammation and immunity, shows two seemingly contradictory functions in tumours. This study aims to investigate the mechanism that regulates IL37 and its role in OSCC progression. Herein, IL37, CD86 and CD206 in OSCC specimens were determined. Hypoxia, MCC950 and siRNA‐Gasdermin D (GSDMD) were utilised to investigate the mechanism of IL37 production and release. Animal experiments were established to examine the role of IL37 in OSCC growth in vivo. We found the levels of IL37 are elevated in OSCC tissues compared with normal oral mucosa. In cell experiments, hypoxia was proved to be a vital facilitator in IL37 expression and release. Mechanically, hypoxia promoted IL37 expression through the activation of NACHT–LRR–PYD‐containing protein 3 (NLRP3) inflammasome, and promoted IL37 release via GSDMD. Furthermore, IL37 levels in OSCC specimens are positively correlated with the number of M2 macrophages, but negatively with M1. Further studies revealed IL37 facilitated OSCC progression via promoting macrophage polarization from M1 to M2 and enhancing tumour cell proliferation. Thus, IL37 could be a promising target for OSCC treatment in the future.

## 1 INTRODUCTION

Oral cancer ranks 18th among the most common tumours worldwide, with approximately 377 thousand new cases and nearly 178 thousand deaths reported in 2020.^1^ Oral squamous cell carcinoma (OSCC) accounts for 90% of all oral cancer cases.^2^ The current primary treatment approach for OSCC patients involves surgical resection, followed by postoperative radiotherapy or postoperative chemoradiotherapy as necessary.^3^ Despite significant advancements in treatment over the past four decades, both tumour recurrence and overall survival rates remain unsatisfactory.^4^ Therefore, it is imperative to further explore the molecular mechanisms underlying the development of OSCC to uncover more effective treatment strategies.

Interleukin 37 (IL37) is a member of the IL1 family and is recognized as a natural inhibitor of immune and inflammatory responses.^5^ In recent years, numerous studies have highlighted the significant role of IL37 in various types of tumours.^6^, ^7^ A prior investigation focused on OSCC revealed a connection between IL37 and the risk of malignant transformation in premalignant lesions.^8^ Specifically, it was found that serum IL37 levels were inversely correlated with the percentage of CD3^+^CD8^+^ T cells in OSCC patients, suggesting that IL37 may exert regulatory control over the adaptive immune response and subsequently impact the progression of OSCC.^9^ However, the precise role of IL37 in OSCC and the mechanisms through which IL37 influences OSCC progression require further investigation.

Tumour‐associated macrophages (TAMs) represent a critical component of the tumour microenvironment and can be classified into two primary phenotypes: M1, which exert an anti‐tumour effect, and M2 macrophages, associated with a pro‐tumour effect.^10^ Research by Kazumasa et al. has demonstrated that TAMs infiltrating OSCC tend to exhibit the M2 phenotype, suggesting their potential involvement in OSCC development.^11^ Another study conducted by Gunassekaran et al. suggests that reprogramming TAMs to adopt an M1‐like macrophage phenotype can effectively inhibit tumour growth.^12^ Additionally, Xu et al. reported that blocking the polarization of macrophages toward the M2 phenotype resulted in reduced growth, invasion, migration, and angiogenesis in the context of lung cancer.^13^ Therefore, the regulation of macrophage polarization emerges as a promising strategy for the treatment of OSCC.

There have been reports indicating that the balance between M1 and M2 macrophage polarization can vary depending on specific contexts.^10^, ^14^ In a rat model, IL37 was shown to alleviate inflammation in the temporomandibular joint by promoting the transition of M1 macrophages to M2 macrophages.^15^ Similarly, in a mouse model of myocardial infarction, the administration of IL37 was found to modulate macrophage polarization toward the M2 phenotype in the infarcted heart, resulting in a therapeutic effect on myocardial infarction.^16^ The question of whether silencing IL37 could inhibit the progression of OSCC by suppressing M2 macrophage polarization remains an area of ongoing investigation. This research holds promise for understanding the potential role of IL37 in OSCC and its impact on macrophage‐mediated immune responses in the context of tumour progression.

In this study, we conducted an investigation into the expression levels of IL37 in OSCC tissues and observed a notable upregulation of IL37 in both tumour cells and the extracellular matrix. Furthermore, we elucidated the underlying factors contributing to the heightened expression of IL37 in OSCC, explored the mechanisms through which these factors influence IL37 expression and release, and delved into the pivotal role played by IL37 in OSCC progression. Our findings shed light on the intricate relationship between IL37 and OSCC, providing valuable insights into how IL37 impacts the development and advancement of this malignancy.

## 2 MATERIALS AND METHODS

### 2.1 Patients and tissue samples

Thirty primary OSCC tumour samples and sixteen normal oral mucosa samples were collected from OSCC patients at the Stomatological Hospital Oral and Maxillofacial Surgery Department of Chongqing Medical University. All OSCC specimens underwent pathological examination for confirmation. None of these patients had received pre‐operative chemotherapy or radiotherapy. The study protocols received approval from the hospital's Ethics Committee. The clinical parameters of the OSCC patients are presented in Table 1.

**TABLE 1:** Correlation between IL37 expression and clinicopathological features in patients with OSCC.

Characteristics	*n*	IL37 expression			*p*‐value
		Weak	Moderate	High	
Age (years)					
≤60	8	2	4	2	0.8892
>60	22	5	13	4	
Gender					
Male	20	5	11	4	0.9508
Female	10	2	6	2	
Differentiation					
Well	14	3	9	2	0.0666
Medium	14	4	8	2	
Poor	2	0	0	2	
Location					
Gingiva	4	1	3	0	0.2625
Tongue	8	1	6	1	
Buccal mucosa	10	2	5	3	
Floor of mouth	6	1	3	2	
Palate	2	2	0	0	
T stage					
T1–T2	15	4	7	4	0.5120
T3–T4	15	3	10	2	
N stage					
N0	21	6	12	3	0.3737
N1–N3	9	1	5	3	
Clinical TNM stage					
I	2	1	1	0	0.9278
II	7	2	4	1	
III	6	1	4	1	
IV	15	3	8	4	

*Note*: *p*‐values are based on chi‐squared test. *p* < 0.05 indicates a significant association among the variables.

### 2.2 Cell culture and macrophage induction

The human OSCC cell lines HN6 and SCC15 were generously provided by the Chongqing Key Laboratory of Oral Diseases and Biomedical Sciences and cultured in DMEM high glucose medium (HyClone, USA). THP‐1 cells were procured from Procell Life Science & Technology Co., Ltd. (Procell, China) and maintained in RPMI 1640 medium (HyClone, USA). All cell media were further supplemented with 10% fetal bovine serum (BI, Israel) and 1% penicillin/streptomycin. Cells were cultured in a standard atmosphere at 37°C, comprising 20% O_2_, 5% CO_2_, and 75% N_2_. For hypoxia treatment, cells were placed in a hypoxia incubator (Thermo Scientific, USA) with reduced oxygen tension, specifically 1% O_2_, 5% CO_2_, and 94% N_2_, at 37°C for the specified duration.

THP‐1 cells were stimulated with 100 ng/mL phorbol 12‐myristate 13‐acetate (Sigma‐Aldrich) for 24 h to induce M0 macrophages. Next, M0 macrophages were treated with 500 ng/mL LPS (Sigma‐Aldrich) and 50 ng/mL IFN‐γ (Sinobiological, China) for 24 h to induce M1 phenotype cells. M1 macrophages were then exposed to 1 ng/mL rhIL37 (Sinobiological, China) for 24 h to induce repolarization of M1 to M2 phenotype.

### 2.3 shRNA transfection

Control shRNA lentiviral particles (mock) and IL37 shRNA lentiviral particles (shIL37) were provided by Tsingke Biotechnology (Beijing, China). Following the reagent instructions, we determined the appropriate MOI values and polybrene concentration (Beyotime, China) for transfection into HN6 cells through preliminary experiments. Subsequently, we employed shRNA to transfect HN6 cells using conditions involving an MOI of 10 and a polybrene concentration of 2 μg/mL. Ten hours post‐transfection, the cells were transferred to fresh medium and cultured for an additional 2 days. Afterward, they were subjected to treatment with 1 μg/mL puromycin (Beyotime, China) for 7 days to select for stably transfected cells. Subsequent to this selection process, we assessed the transfection efficiency in preparation for further investigations.

### 2.4 siRNA transfection

Non‐silencing control siRNA (siRNA‐control) and GSDMD‐targeting siRNA (siGSDMD) were procured from Tsingke Biotechnology (Beijing, China). HN6 cells were transfected with siRNA for a duration of 6 h using EntransterTM‐R4000 transfection reagent (Engreen Biosystem, China), following the manufacturer's guidelines. Following the transfection, the cells were allowed to incubate in fresh medium for an additional 12 h. Subsequently, they underwent a 24‐h treatment with hypoxia.

### 2.5 RNA extraction and qPCR

To assess the mRNA expression of a specific gene, we extracted total RNA from cells or tissues using Trizol reagent (Takara, Japan). Subsequently, we performed cDNA synthesis using the PrimeScript RT Master Mix (Takara, Japan). For quantitative PCR (qPCR), we utilized SYBR Premix Ex Taq II (Takara, Japan). Either β‐actin or Gapdh was employed as an internal control reference. The specific primers utilized in this experiment were custom‐synthesized by Takara Bio, and the sequence of each primer can be found in Table 2.

**TABLE 2:** Primer sequences for RT‐qPCR.

Gene	Primer sequence (5′‐3′)
Gapdh	Forward: CTTTGGTATCGTGGAAGGACTC Reverse: GTAGAGGCAGGGATGATGTTCT
β‐Actin	Forward: AGAAAATCTGGCACCACACCT Reverse: GATAGCACAGCCTGGATAGCA
IL37	Forward: TTGCATTAGCCTCATCCTTGA Reverse: GGCGTGCTGATTCCTTTTG
NLRP3	Forward: CGAAGTGGGGTTCAGATAATG Reverse: TCGTGTGTAGCGTTTGTTGAG
Caspase1	Forward: CTCAGGCTCAGAAGGGAATGTC Reverse: TGCGGCTTGACTTGTCCATT
Gasdermin D	Forward: GGAAACCCCGTTATAAGTGTGT Reverse: TGCCCTGTATCTGCCCATC
IL1β	Forward: GAAATGATGGCTTATTACAGTGGC Reverse: TTGCTGTAGTGGTGGTCGGAG
HIF1α	Forward: ATGAAGTGTACCCTAACTAGCCG Reverse: GTTCACAAATCAGCACCAAGC
CD86	Forward: GTTTCATTCCCTGATGTTACGAG Reverse: GAGAAAGGTGAAGATAAAAGCCG
iNOS	Forward: CAGGACTCACAGCCTTTGGAC Reverse: TGGATGTCGGACTTTGTAGATTC
CXCL10	Forward: CCGGAATTCGAGCCTACAGCAGAGGAACC Reverse: CCGCTCGAGTTTGCTCCCCTCTGGTTTTA
CD206	Forward: TGATACCTGCGACAGTAAACGA Reverse: CTTGCAGTATGTCTCCGCTTC
CD163	Forward: TCGCTCATCCCGTCAGTCA Reverse: CCGCTGTCTCTGTCTTCGCT
IL10	Forward: CAAGACCCAGACATCAAGGCG Reverse: GCATTCTTCACCTGCTCCACG

### 2.6 Western blot

Cells and fresh tissues were lysed using RIPA buffer (Beyotime, China) supplemented with phosphatase and protease inhibitors (Beyotime, China). The total protein concentration was determined using the BCA Protein Quantitative Kit (Beyotime, China). Equal amounts of protein were loaded onto sodium dodecyl sulfate (SDS)‐polyacrylamide gel electrophoresis (PAGE) and subsequently transferred onto a polyvinylidene difluoride membrane (Millipore, USA). These membranes were then blocked with 5% BSA in TBST for 2 h, followed by an overnight incubation with primary antibodies at 4°C. After the overnight incubation, the membranes were incubated with secondary antibodies for 1 h at room temperature. The protein bands were visualized using an ECL reagent (Beyotime, China) and captured using a Bio‐Rad Gel Imaging System (Bio‐Rad, USA).

### 2.7 Elisa

Cells with identical initial cell counts were cultured in uniform volumes of medium, and the growth medium supernatants were harvested once the cells reached 90% confluence. The concentration of extracellular IL37 in these growth medium supernatants was determined using a commercially available human IL37 ELISA kit (Bioss, China), following the manufacturer's instructions.

### 2.8 Immunofluorescence staining

The cells were fixed using 4% paraformaldehyde for 15 min at room temperature, followed by permeabilization using PBST (PBS with 0.2% Triton X‐100) for 15 min. Subsequently, they were blocked with 10% goat serum (Bioss, China) for 30 min and incubated overnight at 4°C with anti‐CD86 antibody (Bioss, China) or anti‐CD206 antibody (Abcam, UK). After three washes, the cells were incubated with Alexa Fluor 488‐conjugated secondary antibody (Bioss, China) for 1 h. To visualize the cell nuclei, 4′,6‐diamidino‐2‐phenylindole (Bioss, China) was used as a counterstain. The images were captured using a fluorescent microscope (Axio Observer7, Carl Zeiss) and analysed using ZEN Blue software (Carl Zeiss).

### 2.9 Cell proliferation assay

Cell proliferation was assessed using CCK‐8 (MedChemExpress, China), following the manufacturer's instructions. In brief, cells were seeded into 96‐well plates at a density of 5 × 10^3^ cells per well and cultured for the specified time intervals. Subsequently, 10 μL of CCK‐8 solution was added to the culture medium in each well. After incubating for 1 h, the optical density (OD) values were measured using a microplate reader (Bio‐Rad, CA) at a wavelength of 450 nm. Each time point was replicated in triplicate, and the experiment was independently conducted three times.

### 2.10 Flow cytometry

To determine the apoptosis rate of HN6 cells, a total of 5 × 10^5^ cells were quantified utilizing the Annexin V‐APC/PI apoptosis detection kit (KeyGene, China), following the manufacturer's instructions. Subsequently, CytoFLEX (Beckman CytoFlex, USA) was employed to quantify the apoptotic cells. The apoptosis levels of cells in various experimental groups were expressed as the apoptosis rate (%).

### 2.11 Wound healing assay

Cells were seeded in a six‐well plate at a concentration of 5 × 10^5^ cells/mL and cultured until they reached 95%–100% confluence. Subsequently, a 200 μL sterile pipette tip was used to create a uniform‐width scratch in the cell monolayer. The closure of the wound was observed under a microscope at 0, 6, and 12 h after scratching. The wound areas were quantified using ImageJ software (NIH, USA), with the wound area at 0 h considered as the baseline (100%). This allowed for the assessment of the relative wound closure over time.

### 2.12 Haematoxylin and eosin (H&E) staining

Tissue samples were initially fixed in 4% paraformaldehyde overnight, followed by overnight flushing with running water. They were then subjected to dehydration using xylene and alcohol, embedded in paraffin, and sliced into sections with a thickness of 4 μm. These sections were subsequently stained with haematoxylin and eosin (Solarbio, China), following the manufacturer's provided instructions. Finally, the stained sections were digitally scanned (Olympus, Japan) to obtain images for further analysis.

### 2.13 Immunohistochemistry

Paraffin‐embedded samples were cut into 4 μm‐thick sections for immunohistochemistry. The sections were incubated for 2 h at 65°C, followed by deparaffinization with xylene and alcohol. The prepared sections were pre‐treated using heat mediated antigen retrieval with sodium citrate buffer for 20 min at 95°C, incubated in 3% H_2_O_2_ for 15 min, blocked with 10% goat serum (Bioss, China) for 30 min at 37°C. Next, the sections were incubated with primary antibody against specific protein overnight at 4°C. For the negative control, PBS was added instead of primary antibody. The next day, the sections were incubated with HRP‐conjugated secondary antibody for 1 h at room temperature. Diaminobenzidine (Bioss, China) was used as the chromogen and haematoxylin was used as a counterstain. Finally, the sections were dehydrated in ethanol, cleared in xylene, and covered with coverslips. Images were obtained by using Olympus VS200 digital scanner (Olympus, Japan). Then we adopted the following criteria to score staining: staining intensity: 0‐no detectable, 1‐light yellow, 2‐medium yellow, 3‐deep yellow, 4‐brown; and staining proportion: 1 (≤10%), 2 (10%–50%), 3 (50%–80%), 4 (≥80%).^17^ The final staining score was calculated by multiplying staining intensity score and staining proportion score. We defined 0 as negative, 1–4 as weak, 5–11 moderate, and > 11 as high.

### 2.14 Animal experiments

Specific‐pathogen‐free female, 6‐week‐old, Balb/c nude mice were purchased from Byrness Weil biotech Ltd., Chongqing, China. These mice were housed in an animal facility maintained at a temperature of 26°C–28°C, with a 12/12‐h light/dark cycle and humidity ranging from 40% to 60%. To establish the tumour growth model, HN6 cells (1.0 × 10^6^ cells in 100 μL PBS) were subcutaneously injected into the dorsal right flank of each mouse. After injection, we measured the tumour diameters every 3 days for a total of 21 days. Tumour volume (mm3) was calculated using the formula: volume = (shortest diameter)^2^ × (longest diameter) × 0.5. At 21 days post‐injection, the mice were humanely euthanized, and the subcutaneous tumours were collected for further analysis. All experimental procedures were conducted in accordance with the guidelines and were approved by the Biomedical Ethics Committee of Chongqing Medical University.

### 2.15 Statistical analysis

All cell experiments were performed at least three times independently, and each experimental group had three parallel groups. Animal experiments were performed with six nude mice in each group. GraphPad Prism 9.0 was used for statistical analysis and plotting. The data were statistically analysed by using *t*‐test, one‐way ANOVA or chi‐squared test. Results were presented as the mean ± standard error of the mean (SEM). Data with p‐values <0.05 were considered statistically significant.

## 3 RESULTS

### 3.1 IL37 is highly expressed in tumour cells and extracellular matrix of OSCC

**FIGURE 1:**
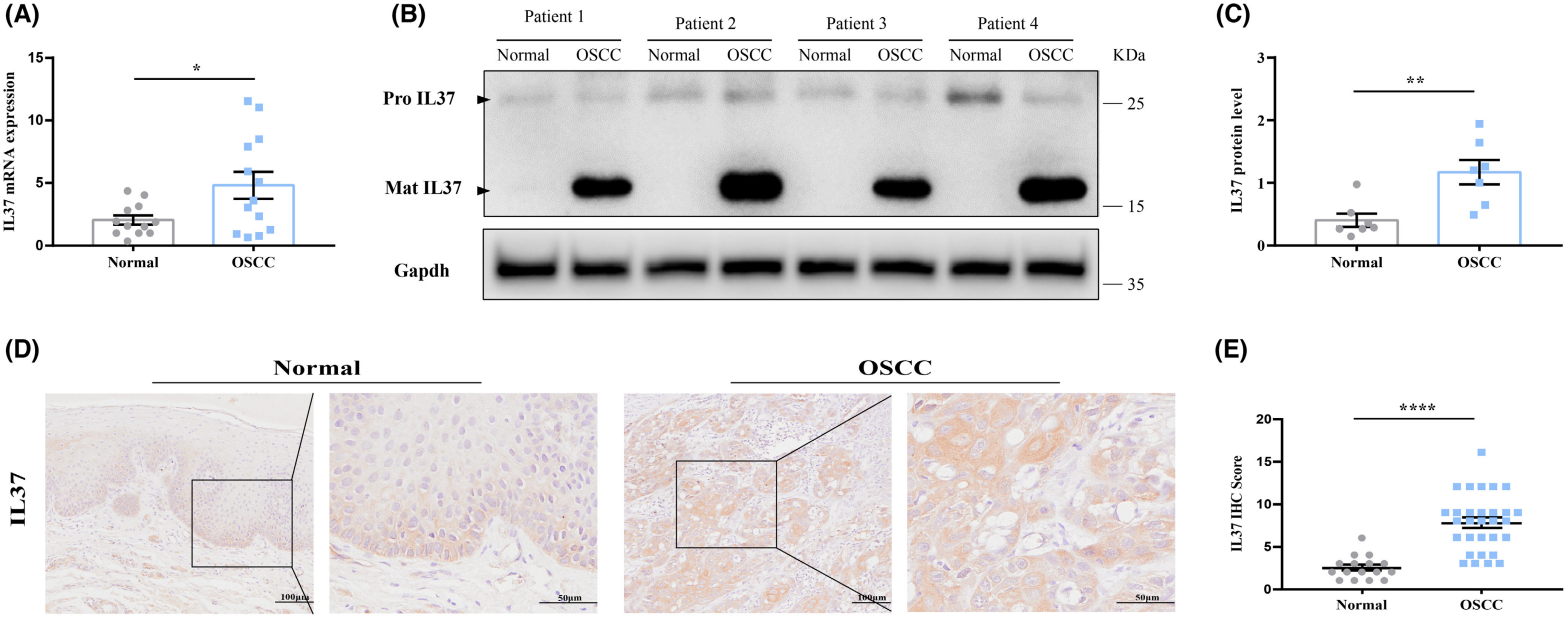
Compared with normal oral mucosa, IL37 expression level was elevated in OSCC specimens. (A) Relative IL37 mRNA levels in normal oral mucosa and OSCC specimens. (B) The expression of IL37 in normal oral mucosa and OSCC specimens detected by western blot. (C) Grey value analysis of western blot results. (D) IL37 expression in normal oral mucosa and OSCC specimens as shown by immunohistochemical staining. (E) Comparison of the staining score between normal oral mucosa and OSCC specimens. *p < 0.05; **p < 0.01; ****p < 0.0001; ns, not significant.

The investigation into IL37 expression in tumour and normal oral mucosa of OSCC patients yielded significant findings. Both IL37 mRNA and IL37 protein levels were found to be elevated in tumour tissues when compared to normal oral mucosa (Figure 1A–C). Furthermore, it was observed that mature IL37 (Mat‐IL37) was the predominant form of IL37 present in OSCC tissues. Immunohistochemical staining revealed that OSCC specimens exhibited higher levels of IL37 in comparison to normal oral mucosa. IL37 was prominently expressed within the tumour cells and the extracellular matrix of tumour tissues (Figure 1D,E). These results strongly suggest that IL37 levels are elevated not only in tumour cells but also within the extracellular matrix of OSCC.

### 3.2 Hypoxia promotes the expression and release of IL37 in OSCC cells

**FIGURE 2:**
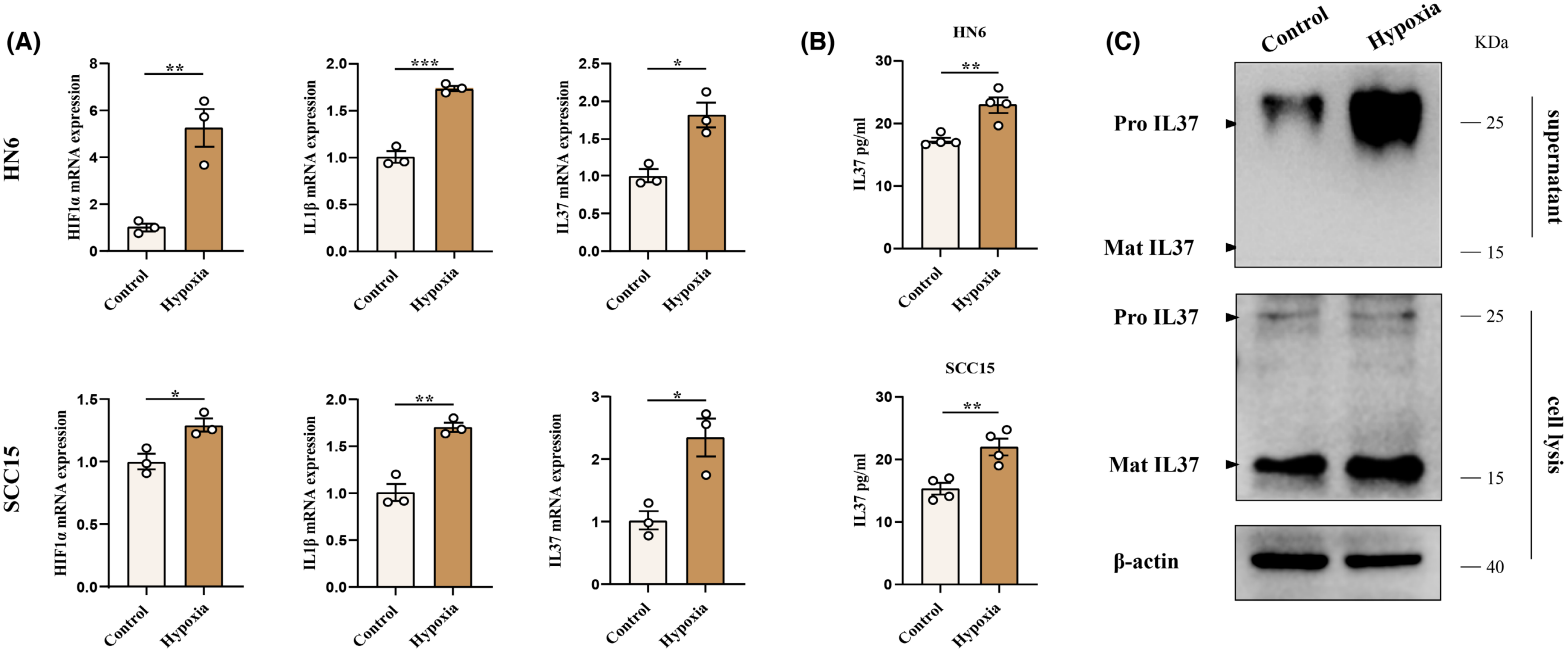
Hypoxia promotes the expression and release of IL37 in OSCC cells. (A) Quantification of mRNA levels of HIF1α, IL1β and IL37 in HN6 cell line (left) and SCC15 cell line (right) under normal or hypoxic condition. (B) The concentration of IL37 in OSCC cell supernatant was detected by Elisa. (C) The levels and form of IL37 in cellular lysis and supernatant were analysed by western blot. *p < 0.05; **p < 0.01; ***p < 0.001; ns, not significant.

To investigate the potential impact of a hypoxia‐induced inflammatory microenvironment on IL37, OSCC cells were exposed to hypoxia. The results revealed a significant increase in the mRNA levels of hypoxia‐inducible factor‐1‐α (HIF1α) and IL1β under hypoxic conditions. Furthermore, there was a notable increase in the levels of IL37 mRNA (Figure 2A), suggesting a potential association between IL37 expression and the presence of an inflammatory environment induced by hypoxia. Additionally, hypoxia was found to enhance the concentration of IL37 in the supernatant (Figure 2B). Western blot analysis demonstrated that hypoxia led to an increase in the levels of Mat‐IL37 within the cell lysate and facilitated the release of IL37 precursor (Pro‐IL37) into the extracellular space (Figure 2C). These findings indicate that a hypoxia‐induced inflammatory microenvironment promotes the expression and release of IL37 in OSCC cells.

### 3.3 Hypoxia promotes IL37 expression via NLRP3 inflammasome activation and IL37 release via Gasdermin D

**FIGURE 3:**
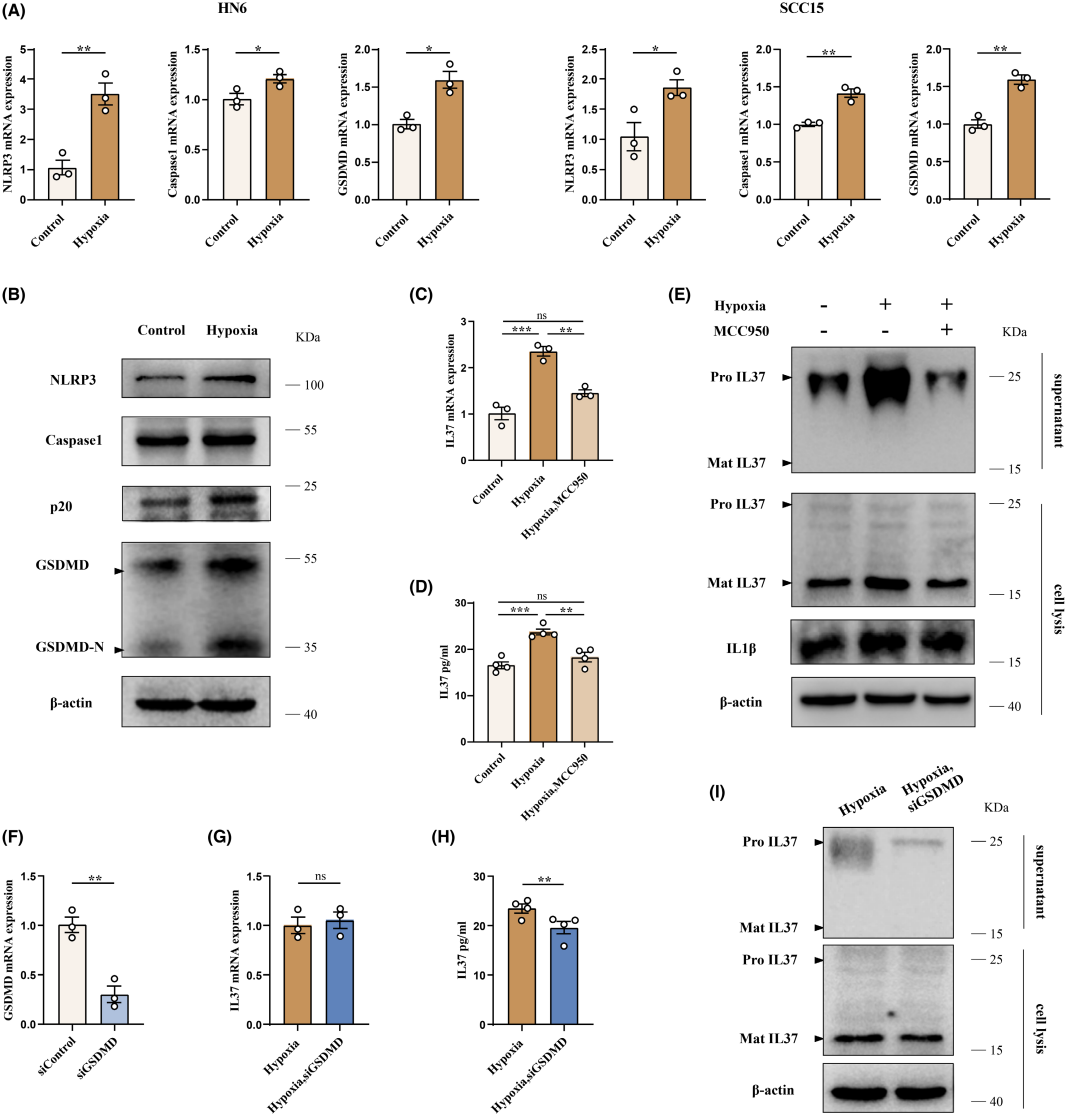
Hypoxia promotes the expression and release of IL37 via NLRP3 inflammasome/GSDMD pathway. (A) Quantification of mRNA levels for NLRP3, Caspase1 and GSDMD in HN6 cell line (left) and SCC15 cell line (right) under normal or hypoxic condition. (B) Protein levels of NLRP3, Caspase1, Caspase p20, GSDMD and GSDMD‐N were determined by western blot. (C–E) OSCC cells were treated with or without MCC950 under hypoxic condition. (C)The relative mRNA levels of IL37 in OSCC cells were analysed. (D) The concentration of IL37 in OSCC cell supernatant was measured by Elisa. (E) The levels of IL37 and IL1β as shown by western blot. (F) The relative mRNA levels of GSDMD in OSCC cells transfected with siRNA‐Control or siRNA‐GSDMD was analysed. (G–I) After transfection with or without siRNA‐GSDMD, OSCC cells were cultured in hypoxic condition. (G) The relative mRNA levels of IL37 in OSCC cells was detected. (H) The concentration of IL37 in OSCC cell supernatant was measured by Elisa. (I) The levels and form of IL37 in cellular lysis and supernatant as shown by western blot. *p < 0.05; **p < 0.01; ***p < 0.001; ns, not significant.

To explore the mechanism underlying how the hypoxia‐induced inflammatory microenvironment promotes the expression and release of IL37, we assessed the expression levels of NLRP3, Caspase1, and Gasdermin D. The findings demonstrated that hypoxia led to an increase in the mRNA and protein expression of NLRP3, Caspase1, and GSDMD (Figure 3A,B). Additionally, Western blot analysis revealed an elevation in the levels of Caspase1 p20 (the active form of Caspase1) and GSDMD‐N (the cleaved GSDMD that forms transmembrane pores) in the hypoxia‐exposed group (Figure 3B). For further investigation, OSCC cells were subjected to hypoxia and simultaneously treated with MCC950, a selective NLRP3 inhibitor. The results indicated that MCC950 effectively suppressed the increases in IL37 expression, IL1β expression and IL37 release induced by hypoxia (Figure 3C–E). These findings suggest that the hypoxia‐induced inflammatory microenvironment promotes IL37 expression by activating the NLRP3 inflammasome.

To further investigate the underlying mechanism of IL37 release, we examined the impact of GSDMD on IL37 using siGSDMD. qPCR analysis demonstrated a significant reduction in the expression of GSDMD when treated with siGSDMD (Figure 3F). To delve deeper into this mechanism, HN6 cells were transfected with siGSDMD for 18 h and then incubated in a hypoxic incubator. The results revealed that siGSDMD diminished the increase in extracellular IL37 levels induced by hypoxia, while the expression levels of IL37 mRNA did not change significantly (Figure 3G–I), indicating siGSDMD does not diminish the expression level of IL37, but rather inhibits its release. Overall, these findings suggest that GSDMD is involved in the release of IL37 induced by hypoxia.

### 3.4 IL37 induces repolarization of M1 to M2 macrophages

**FIGURE 4:**
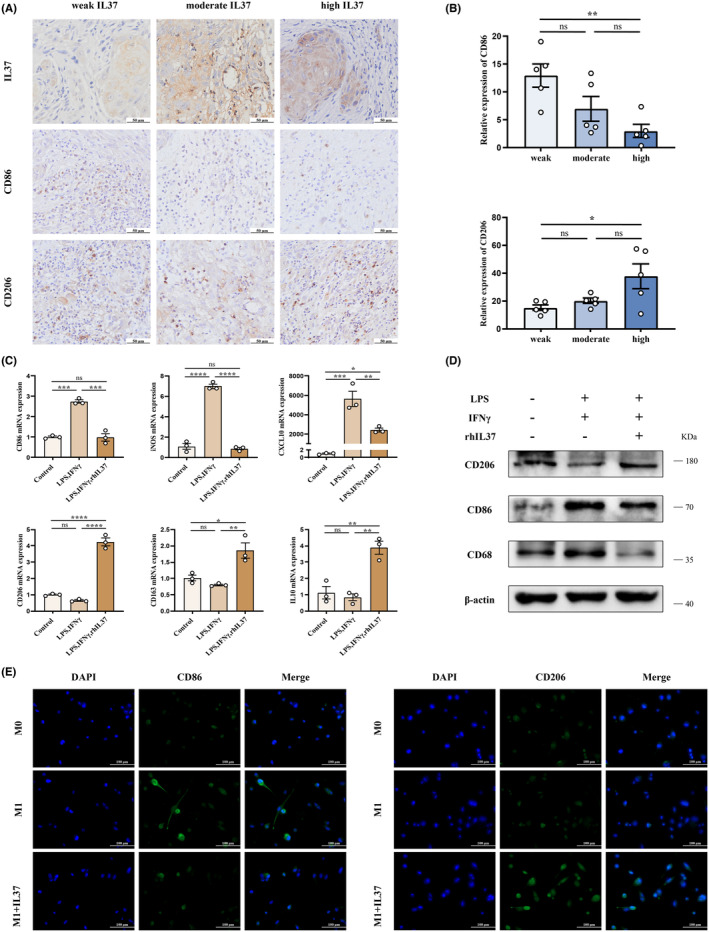
IL37 induces repolarization of M1 to M2 macrophages. (A) The expression of CD86 and CD206 in OSCC as shown by immunohistochemical staining. According to IL37 staining score, OSCC specimens were divided into three groups, weak group, moderate group and high group. (B) Immunohistochemical staining analysis of relative expression of CD86 (up) and CD206 (down) in three groups. (C) After the administration of LPS and IFN‐γ, THP1‐derived macrophages were treated with or without rhIL37. The mRNA levels of M1 and M2 macrophage markers detected by qPCR. (D) The protein levels of CD68, CD86 and CD206 were determined by western blot. (E) Immunofluorescence staining for THP1‐derived macrophages. The green signal represents the staining of CD86 or CD206, and the blue signal represents the DAPI‐stained nuclei. *p < 0.05; **p < 0.01; ***p < 0.001; ****p < 0.0001; ns, not significant.

For investigating the specific role of IL37 in tumour associated macrophage (TAM) polarization, OSCC specimens were divided into three groups: weak group, moderate group and high group according to IL37 IHC score. Then the levels of CD86 and CD206 in tumour tissues in three groups were measured with immunohistochemical staining. The findings revealed a higher prevalence of CD206^+^ macrophages in tumour tissues when compared to CD86^+^ macrophages. Interestingly, the weak group displayed a greater abundance of CD86^+^ macrophages in contrast to the high group. Furthermore, the high group exhibited a significantly higher count of CD206^+^ macrophages compared to the weak group (Figure 4A,B).

To investigate the potential role of IL37 in inducing M2 macrophage polarization, M0 macrophages were first stimulated with LPS and IFN‐γ and then subjected to recombinant human IL37 (rhIL37) induction. The analysis by qPCR revealed that rhIL37 induction resulted in the downregulation of mRNA expression of M1 macrophage markers, including CD86, iNOS, and CXCL10. Conversely, IL37 upregulated the mRNA expression of M2 macrophage markers, such as CD206, CD163, and IL10 (Figure 4C). These findings were further confirmed at the protein level through Western blot analysis (Figure 4D) and immunofluorescence staining (Figure 4E). This suggests that IL37 plays a role in polarizing macrophages from an M1 to M2 state.

### 3.5 IL37 knockdown inhibited the proliferation and migration of OSCC cells in vitro

**FIGURE 5:**
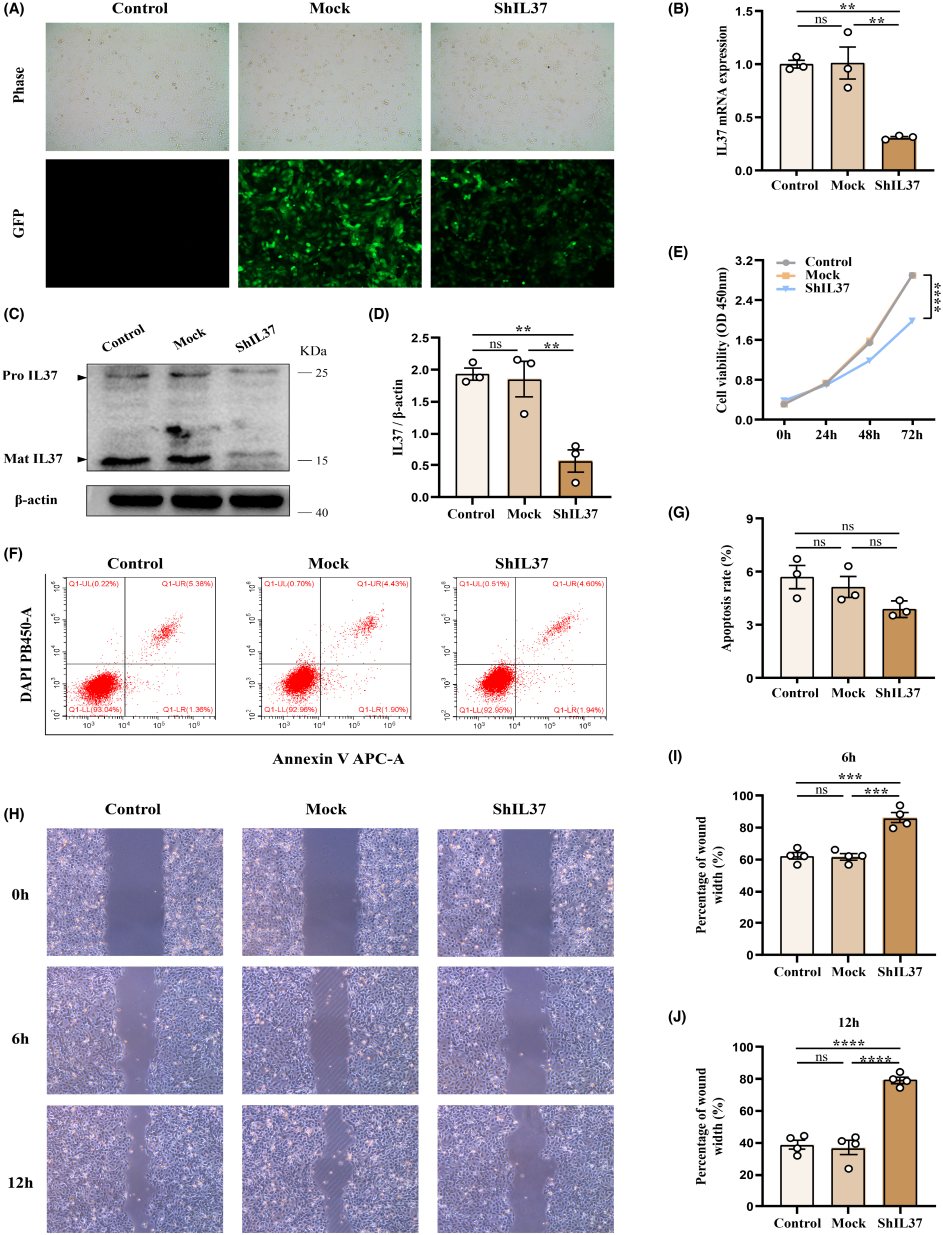
IL37 knockdown inhibits proliferation and migration of OSCC cells in vitro. (A–D) The identification of shRNA interference efficiency. HN6 cells were transfected with shRNA‐Control or shRNA‐IL37. Control cells received no transfection. (A) Bright‐field and fluorescence images of HN6 cells captured by inverted fluorescence microscopy. (B) The quantification of IL37 mRNA levels in HN6 cells. (C) The protein levels of IL37 as shown by western blot. (D) Quantitative analysis of western blot data. (E) CCK8 assays were performed to analyse cell proliferation at 24, 48, 72 h. (F, G) Flow cytometric analysis of cell apoptosis. The sum of number in the right quadrant of each panel represents the apoptosis rate. (H–J) The migration of OSCC cells was determined by wound healing assays. The wound with were calculated at oh, 6 h, 12 h. **p < 0.01; ***p < 0.001; ****p < 0.0001; ns, not significant.

To elucidate the biological impact of IL37 on OSCC cells, control shRNA (mock) and shIL37 constructs were established and transfected into HN6 cells. The efficiency of interference was assessed, and a significant reduction in IL37 expression was observed in HN6 cells following shIL37 transfection (Figure 5A–D). Subsequently, CCK‐8 assays were conducted, revealing a marked decrease in cell proliferation in the shIL37 group compared to the control and mock groups (Figure 5E). However, when we measured the cell apoptosis rate of HN6 cells using flow cytometry, the analysis results showed no significant difference among the control, mock, and shIL37 groups (Figure 5F,G). Furthermore, wound healing assays were performed, demonstrating that the migration rate of the shIL37 group was significantly slower than that of the control and mock groups (Figure 5H–J). In summary, these data strongly suggest that IL37 plays a critical role in promoting OSCC tumorigenesis by facilitating cell proliferation and migration.

### 3.6 IL37 knockdown inhibited OSCC progression by inhibiting macrophage repolarization of M1 to M2, as well as the proliferation of tumour cells

**FIGURE 6:**
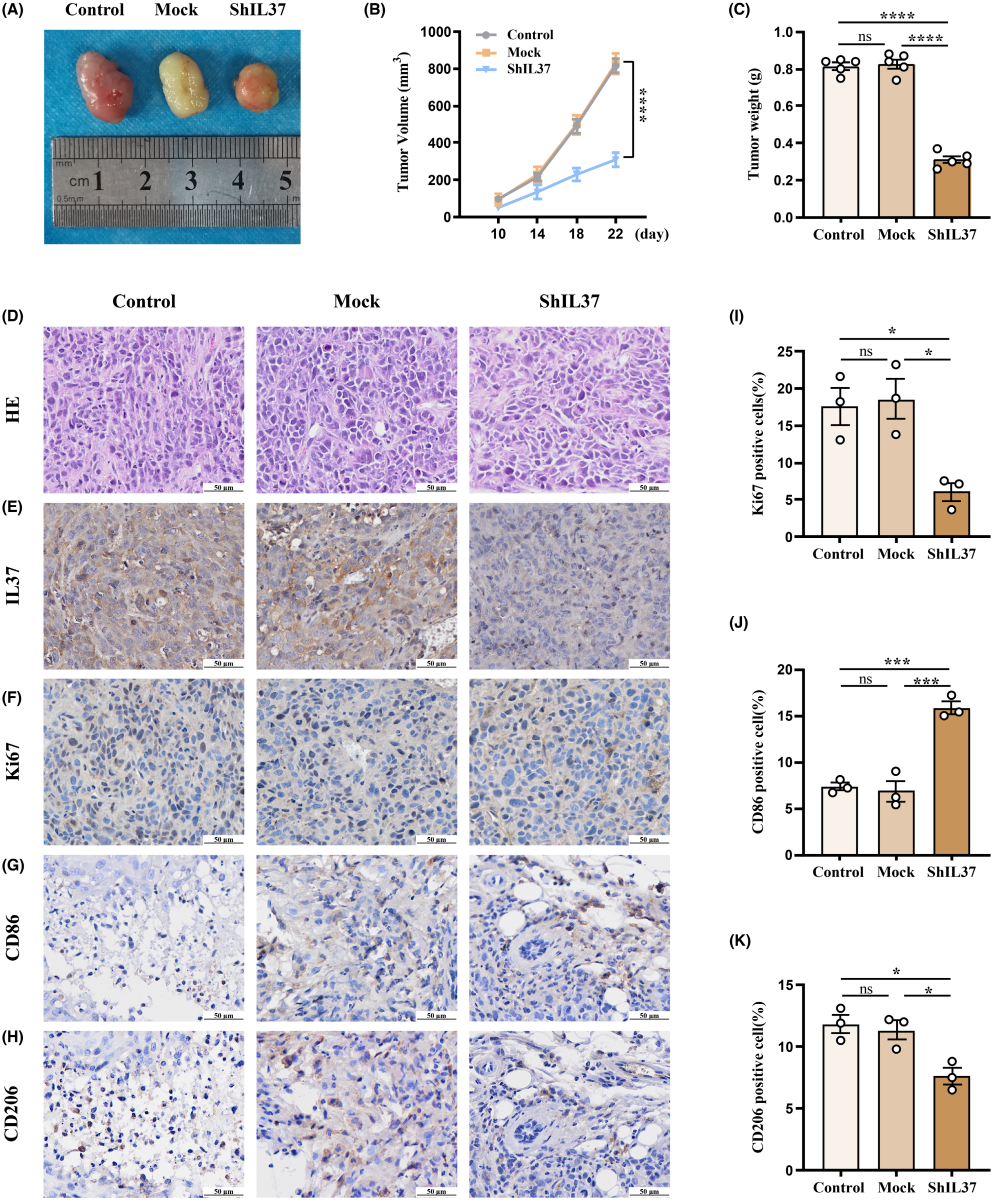
IL37 knockdown inhibits OSCC progression by suppressing macrophage repolarization of M1 to M2 and the proliferation of tumour cells. HN6 cells and HN6 cells transfected with shRNA‐Control or shRNA‐IL37 were implanted into nude mice. (A) Representative image of tumours derived from mice in each group. (B) Tumour volumes of each group at different time points. (C) Tumour weights of each group after resection. (D–H) Representative images of tumour specimens stained for (D) HE and immunostained for (E) IL37, (F) Ki67, (G) CD86 and (H) CD206 in each group. (I) Quantification of Ki67 positive cells. (J, K) Quantification of macrophage markers in non‐tumour cells. (J) CD86 positive cells. (K) CD206 positive cells. *p < 0.05; ***p < 0.001; ****p < 0.0001; ns, not significant.

To investigate the impact of IL37 on OSCC progression in vivo, an OSCC xenograft model was established in Balb/c nude mice. Parental cells (control), control shRNA‐transfected cells (mock), and IL37 RNA‐transfected cells (shIL37) were subcutaneously implanted into the dorsal right flank of each mouse (Figure 6A). Tumour volumes were calculated, and tumour weights were recorded. The data demonstrated a significant reduction in tumour volume, size, and weight in the shIL37 group compared to those in the control and mock groups. In contrast, the tumour growth of the mock group was not significantly different from that of the control group (Figure 6B,C). Within the tumour specimens, the shIL37 group exhibited an increase in CD86 expression (Figure 6G,J) and a decrease in CD206 expression (Figure 6H,K), along with a lower percentage of Ki67‐positive cells (Figure 6F,I), when compared with the control and mock groups. These results collectively suggest that IL37 knockdown inhibits OSCC growth in vivo by suppressing M1‐to‐M2 repolarization and reducing the proliferation of tumour cells.

## 4 DISCUSSION

This study has successfully established a mechanistic connection between two pivotal factors in cancer progression, namely the hypoxic environment and tumour‐associated macrophages (TAMs), through the involvement of IL37 in OSCC. The findings of this study indicate that IL37 accelerates OSCC progression through promoting M2 macrophage polarization and tumour cell proliferation. This study not only offers insights into the high expression of IL37 in OSCC but also sheds light on the role of IL37 in macrophage repolarization and the mechanism of IL37 release. Overall, it provides a novel perspective and potential avenues for the treatment of OSCC.

IL37 is a significant anti‐inflammatory cytokine that plays a crucial role in regulating both innate and adaptive immunity.^18^ While there is a growing body of evidence highlighting the involvement of IL37 in various diseases, including cancer, its specific role in OSCC has remained unclear.^19^ In this study, we noted a high expression of IL37 in OSCC tissue samples, which aligns with previous findings by Lin et al.^8^ Moreover, our study revealed that IL37 was not only highly expressed within tumour cells but also in the extracellular matrix. This suggests that both the production and extracellular secretion of IL37 were enhanced in OSCC. Many have reported that IL37 is upregulated by inflammatory stimulus, and hypoxia is known to be linked to the inflammatory state within the tumour environment.^20^, ^21^ We hypothesized that the upregulation of IL37 in OSCC could be a means of self‐protection for tumour cells against the inflammation induced by the hypoxic tumour environment. Our results indeed showed that hypoxia significantly promoted the expression of IL1β, a pro‐inflammatory cytokine, and concurrently increased IL37 expression. MCC950, an anti‐inflammatory agent by inhibiting NLRP3 inflammasome, decreased level of IL37, which further verified our assumption. Additionally, hypoxia was found to contribute to the release of IL37, specifically in the form of pro‐IL37 into the extracellular space. It is worth noting that extracellular proteases may further cleave pro‐IL37 into its mature form (mat‐IL37).^22^ While the anti‐inflammatory ability of pro‐IL37 is weaker than that of mat‐IL37, both forms are biologically active.^23^ In summary, the elevated expression of IL37 in OSCC tissues appears to be a response by tumour cells to counteract the inflammation induced by the hypoxic tumour microenvironment.

Next, we assessed the expression levels of NLRP3 and Caspase1 in OSCC cells. The results demonstrated that hypoxia activated the NLRP3 inflammasome, suggesting its potential involvement in IL37 expression and release. To further explore this, we pharmacologically inhibited NLRP3 using MCC950. The outcomes indicated that MCC950 attenuated the upregulation of IL37 expression and release induced by hypoxia. This implies that hypoxia promotes IL37 expression and release via the NLRP3 inflammasome. It is noteworthy that previous research has reported that IL37 can alleviate inflammatory diseases by inhibiting NLRP3 inflammasome activation.^16^, ^24^, ^25^ This reciprocal regulation could represent a mechanism that ensures an optimal degree of NLRP3 inflammasome activation. Furthermore, the decrease in IL37 expression accompanied by MCC950 treatment also resulted in reduced IL1β expression, further verifying that elevated IL37 serves as a self‐protective mechanism for tumour cells against inflammation. However, it is important to note that under hypoxic conditions, the increased release of IL37 may be a result of heightened IL37 expression mediated by NLRP3 inflammasome activation. Further investigations are warranted to elucidate the precise role that NLRP3 inflammasome plays in the process of IL37 release.

The activation of the NLRP3 inflammasome leads to the formation of GSDMD pores on the cell membrane.^26^ As previous reports suggest, pro‐IL37 can be secreted into the extracellular space when membrane integrity is compromised.^22^ Recent studies have also indicated that IL1 and IL18 are secreted by macrophages through GSDMD cleavage‐mediated pores.^27^, ^28^ In our study, we observed that in hypoxia‐treated OSCC cells, the increase in IL37 release was accompanied by elevated levels of GSDMD and cleaved‐GSDMD. To further confirm the mechanism, we demonstrated that pro‐IL37 was indeed secreted outside of tumour cells through GSDMD. The levels of extracellular IL‐37 are influenced by the levels of IL37 expression and extracellular secretion pathway. In our study, we found that under hypoxic conditions, silencing GSDMD did not alter the expression level of IL37, but decreased the level of extracellular IL37, indicating extracellular secretion pathway of IL37 is suppressed by silencing GSDMD. These findings strongly suggest that hypoxia promotes IL37 release via the NLRP3/GSDMD pathway. This aligns with the conclusions drawn by Gritsenko et al.^29^ However, it is important to note that in our study, silencing GSDMD did not completely inhibit IL37 release, suggesting the potential existence of alternative secretory pathways for IL37 release. Further investigations are warranted to elucidate these additional mechanisms of IL37 release.

Taking into account the immunosuppressive effects of IL37, our study delved into how IL37 might impact OSCC progression. In OSCC specimens, we observed a significantly higher density of CD206^+^ macrophages in the high IL37 group compared to the weak IL37 group. Conversely, the count of CD86^+^ macrophages in the weak IL37 group was higher than in the high IL37 group. Previous studies have reported that IL37 can mediate disease progression by modulating macrophage polarisation.^15^, ^30^ Building upon these findings, we hypothesized that IL37 could influence OSCC progression by regulating the polarization of M1 and M2 macrophages. Our results showed that the administration of rhIL37 led to a decrease in the expression of M1 macrophage markers while increasing the expression of M2 markers, which is in line with previous studies.^15^, ^16^ In an OSCC xenograft mouse model, the knockdown of IL37 resulted in a slower tumour growth rate, accompanied by an increase in the number of CD86^+^ M1 macrophages and a decrease in CD206^+^ M2 macrophages. These findings collectively suggest that IL37 may potentially promote OSCC progression by inducing the repolarization of M1 macrophages toward an M2 macrophage phenotype.

Considering the high expression of IL37 in OSCC tumour cells, we hypothesized that the abnormal expression of IL37 might have functional significance for the tumour cells themselves. Therefore, we conducted experiments to assess the effects of IL37 on the biological behaviour of OSCC cells. The results of our study indicated that the knockdown of IL37 had no significant effect on cell apoptosis but did inhibit cell proliferation and migration. These findings suggest a pro‐tumour role for IL37 in OSCC cells. This observation was further validated in an OSCC xenograft mouse model, which confirmed the tumour‐promoting ability of IL37. Interestingly, a previous study conducted by Lin et al. also noted an increase in the expression of IL37 from normal oral mucosa to oral leukoplakia and OSCC.^8^ This suggests that IL37 indeed possesses a tumour‐promoting ability, which aligns with our own conclusions. It is important to note that several studies have explored the role of IL37 in various other tumours, including renal cell carcinoma,^31^ lung cancer,^32^ hepatocellular carcinoma,^33^ breast cancer,^34^ and more. These studies collectively demonstrate that IL37 can exhibit anti‐tumour activity by modulating various tumour‐suppressive signalling pathways and regulating both innate and adaptive immunity. The differing functions of IL37 in various tumours may be attributed to the varying sensitivity of different tumour types to IL37, highlighting the complexity of IL37's role in cancer.

**FIGURE 7:**
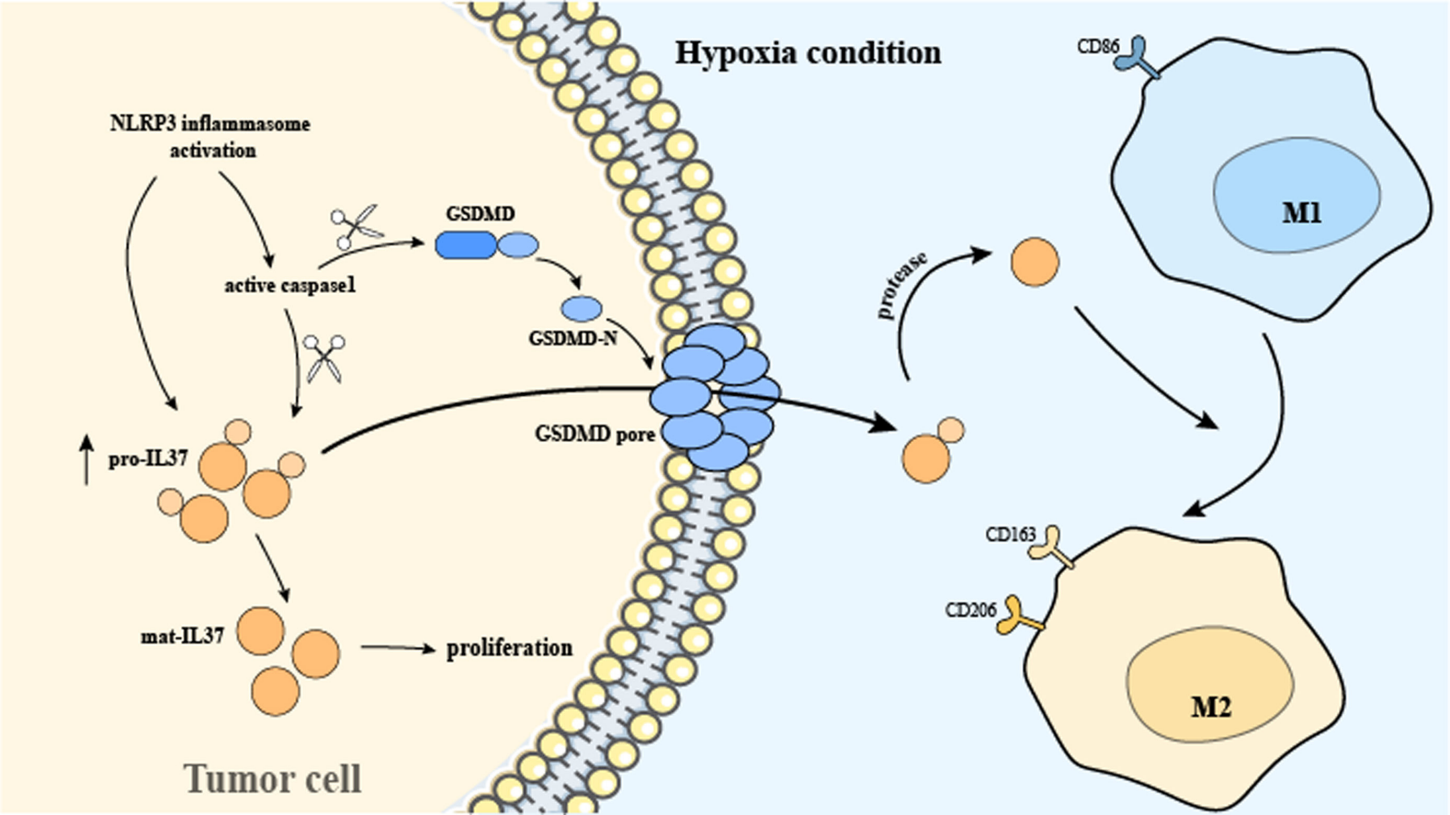
Graphical illustration of the effect of IL37 on OSCC. In tumour cells exposed to a hypoxic environment, the NLRP3 inflammasome is activated, leading to the activation of caspase1 and elevated levels of pro‐IL37. Active caspase1 mediates the processing of pro‐IL37 into mat‐IL37. Besides, active caspase1 also cleaves GSDMD into GSDMD‐N and GSDMD‐C fragments. GSDMD‐N forms GSDMD pores on the cell membrane. On one hand, IL37 promotes tumour cell proliferation. On the other hand, pro‐IL37 is secreted into the extracellular space via GSDMD pores. Subsequently pro‐IL37 is cleaved by extracellular proteases, inducing macrophage polarization from M1 to M2. IL37 facilitates the progression of OSCC through inducing M2 macrophage polarization and tumour cell proliferation.

In conclusion, the findings of this study suggest that the tumour inflammatory environment induced by hypoxia promotes IL37 expression via NLRP3 inflammasome activation, and IL37 release through NLRP3/GSDMD. IL37 promotes the proliferation of tumour cells, leads to the repolarization of M1 macrophage toward M2 macrophage, ultimately accelerating the progression of OSCC (Figure 7). This study may provide new ideas for the treatment of OSCC, while more efforts are still needed.

## AUTHOR CONTRIBUTIONS

**Ying Yan:** Data curation (lead); formal analysis (lead); investigation (lead); methodology (lead); software (lead); validation (lead); visualization (lead); writing – original draft (lead). **Jun Li:** Software (supporting); validation (supporting). **Yungang He:** Methodology (supporting). **Ping Ji:** Resources (supporting). **Jie Xu:** Conceptualization (supporting); funding acquisition (equal); resources (equal); supervision (lead); writing – review and editing (equal). **Yong Li:** Conceptualization (equal); funding acquisition (equal); project administration (lead); resources (equal); writing – review and editing (equal).

## CONFLICT OF INTEREST STATEMENT

The authors declare no conflict of interest.

## Data Availability

The data that support the findings of this study are available from the corresponding author upon reasonable request.
